# Wall Paper

**Published:** 1875-02

**Authors:** 


### WALL PAPER,

WHETHER green or red, is "set" with arsenic, the dust of which is constantly flying about, is breathed into the lungs, and swallowed into the stomach, and thus poisons the blood. Papered walls "set off" an apartment, but it is certain that they collect dust and dirt and are a refuge for worms and insects. No sleeping room ought to be papered; parlors may be, for the paper can be well varnished and kept clean. Paintings and engravings and chromos are more appropriate and more healthful ornaments to a chamber than any paper.

